# Reliability assessment of key equipment for coal gasification using artificial intelligence technology

**DOI:** 10.1371/journal.pone.0350454

**Published:** 2026-06-03

**Authors:** Liping Wu, Ziheng Zhang, Rijia Ding, Wenxin Zhang, Ming Liu

**Affiliations:** 1 School of Management, Heilongjiang University of Science and Technology, Harbin, Heilongjiang, China; 2 School of Management, China University of Mining and Technology- Beijing, Beijing, China; 3 School of Environment and Safety Engineering, Liaoning Petrochemical University, Fushun, Liaoning, China; University of Oklahoma, UNITED STATES OF AMERICA

## Abstract

To address the gap in quantitatively modeling dynamic failure mechanisms for Gasifier lock bucket valve system reliability, this study proposes an innovative method: using backpropagation (BP) neural network to optimize the prior data of dynamic Bayesian network (DBN). Firstly, based on the empirical formula for the number of hidden layer neurons, the original DBN model of the system is adapted to a structurally adaptive BP neural network to calibrate its prior parameters，and the correspondence between the prior distribution of DBN and the input-output functions of the BP network is established. Subsequently, utilizing the core characteristics of BP network, iterative optimization of DBN prior data is achieved through continuous learning of the operating performance of the lock bucket valve system. Next, the optimized DBN model is subjected to dynamic system reliability evaluation using bidirectional inference analysis. The results show that in the positive prediction, the reliability of the system after 300 hours of operation without considering maintenance is only 0.047, which can be improved to 0.302 after incorporating maintenance factors. The reliability of the optimized system is lower than before optimization, and the gap gradually widens over time. Reverse reasoning clearly identifies the weak links in the system as high-pressure coal powder flushing, adhesion between ball seats, internal deformation and wear. Targeted preventive measures can improve the reliability of the system and extend its service life.

## Introduction

The clean and efficient utilization of coal is related to energy security and sustainable economic development. The development of coal chemical technology is a strategic requirement for optimizing the national energy structure and addressing environmental challenges. Its technological research and optimization are of great significance for achieving coordinated development of energy and environment. The essential characteristic of coal chemical industry is to provide various chemical products for the development of national economy through different production processes. Coal gasification technology is a key process in the field of coal chemical industry, which converts solid coal into combustible gas containing carbon monoxide, hydrogen and other components by chemical reaction between coal and gasification agent under high temperature and high pressure through gasifier. The combination of coal gasification industry and traditional industries has derived new technologies and expanded the utilization of resources. It can be seen that the coal gasification industry has important strategic significance in ensuring the stability of the national energy structure and the sustainable development of the energy economy. It is one of the effective technological means to promote high-speed, healthy, and sustainable economic development, truly realizing the comprehensive utilization of resources and the extension of the coal industry chain. The core device of the gasifier is an important part of the entire gasification unit, and the stable operation of the gasifier core system plays a crucial role in ensuring the production safety of the enterprise. As a key equipment of the gasifier, the lock bucket valve is used to control the switch of the ash entering the lock hopper after the gasifier works. If the lock bucket valve fails, it will affect the slag discharge of the gasifier and cause the equipment to malfunction and shut down. Therefore, it is very important to accurately evaluate the reliability of the lock bucket valve system to ensure that the production and operation of the enterprise are not affected by equipment failures and downtime. By evaluating the dynamic reliability of the system, enterprises can promptly identify potential problems and take appropriate measures to reduce risks and improve equipment reliability, thereby enhancing the sustainability of enterprise production.

The precise resolution of issues such as risk assessment, site selection optimization, reliability analysis, and fault diagnosis in key areas such as industrial production, energy development, and equipment maintenance is directly related to ensuring production safety, efficient resource utilization, and stable system operation. It is a core issue in promoting high-quality industrial development. With the rapid iteration of artificial intelligence technology, BP neural network has become an important technical tool for solving complex uncertainty problems due to its excellent nonlinear mapping ability and adaptive learning characteristics. It has been widely applied in various related research fields and has achieved significant results. For example, Xu al. combined fuzzy comprehensive evaluation with BP neural network to construct an electrical fire risk assessment model, providing scientific support for fire prevention and control decisions [1]; Wei et al. built a coal underground gasification site selection model based on fuzzy evaluation BP neural network, effectively improving the rationality and accuracy of the site selection plan [2]; In the field of pipeline engineering, Jing et al. have conducted research on the reliability of corroded pipelines through the integration of BP neural networks and Monte Carlo methods, providing key technical support for pipeline safety operation and maintenance [3]; Xie et al. used GA to improve BP neural network and achieved precise localization of vibration fatigue damage in offshore oil and gas pipelines [4]. In the field of equipment fault diagnosis, relevant achievements have also been fruitful: Shi et al. used an improved BP neural network to complete the diagnosis of synchronous belt faults in machine tools [5], Xie et al. applied BP neural networks to fault recognition of scintillation detectors [6], and Chen et al. combined improved genetic algorithms with BP neural networks to conduct research on bearing fault diagnosis [7], all of which greatly improved the efficiency and accuracy of fault diagnosis. These studies have fully verified the adaptability and effectiveness of BP neural networks in solving complex problems, but further exploration and improvement are needed for the unique requirements of different application scenarios in terms of model structure optimization and strategy adaptability.

As a core equipment in the field of energy production, the stable operation of gasification furnaces directly affects energy production efficiency and safety, and its related risk assessment and optimization research have attracted much attention. In recent years, many scholars have conducted risk assessments on gasification furnaces. For example, so as to address the problems of zero fault data and dynamic faults in gasification systems, Liu et al. took the lock valve system of gasification furnaces as the research object, proposed a method based on dynamic Bayesian networks (DBN), and combined it with Monte Carlo simulation for reliability analysis. This method validly addresses the issue of zero fault data and improves the accuracy of prediction and inference through structural learning and parameter learning [8]. A method based on the integration of Bayesian network and ladder intuitionistic fuzzy number similarity aggregation method (TpIFN-SAM) was proposed by Liu et al. for the study of coal gasification furnace. The research included constructing Bayesian network, collecting expert opinions, aggregating opinions, deblurring processing, calculating system failure probability, and diagnosing key nodes. The failure risk assessment of the gasification furnace was carried out [9]. In order to solve the domino effect problem in the coal gasification process, Zhao et al, taking coal gasification process as the research object, a method combining Fuzzy Analytic Hierarchy Process (FAHP) and Bayesian Network (BN) was proposed to evaluate the impact of domino effect [10]. Gao et al proposed a method that combines dynamic bow tie model (DBT) and dynamic Bayesian network (DBN) model to conduct dynamic quantitative analysis on gasifier overheating accidents [11]. However, existing research based on DBN still has significant limitations: traditional methods rely on expert knowledge to determine DBN prior parameters, which are subjectively limited by expert experience and the influence of parameter allocation differences between different experts. The resulting parameters are highly subjective and difficult to ensure the accuracy of evaluation results, which restricts the depth and effectiveness of gasifier related system optimization.

Based on this, this article focuses on the technical optimization and sustainable development needs of the gasifier lock valve system, and proposes an innovative solution based on backpropagation (BP) neural network optimization of DBN prior data [12]. By constructing a structurally adapted BP neural network and utilizing its powerful nonlinear fitting and self-learning capabilities, iterative optimization of DBN prior data can be achieved, thereby improving the accuracy of dynamic reliability assessment; At the same time, combined with bidirectional reasoning analysis, clarify the reliability change trend and weak links before and after system optimization, providing scientific basis for equipment lifecycle management. The development of this study can provide a practical path for the coordinated promotion of “technological upgrading green development” of energy production core equipment. It has important theoretical and practical significance for promoting the transformation of the energy industry towards a low-carbon and efficient sustainable development model.

## Optimizing DBN method based on BP neural network

### DBN Model

**Fig 1:**
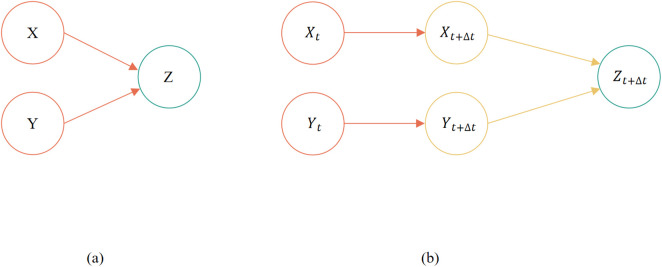
Instance of dynamic Bayesian model.

The DBN model is based on probabilistic networks, combining the existing static Bayesian network ${\text{B}}_{\text{0}}$ with temporal information to form a new stochastic model capable of processing temporal data. Define a DBN network as $\left({\text{B}}_{\text{0}},{\text{B}}_{\to }\right)$, with initial network ${\text{B}}_{\text{0}}$ specifying the joint probability distribution P[X(0)] under initial state X(0); Transfer network ${\text{B}}_{\to }$ specifies the transition probability P{X(t+Δt)|X(t)} of the variable set state from time t to time t+Δt. The dynamic Bayesian network is shown in Fig 1.

Dynamic Bayesian networks can be extended from multiple monolithic time periods.

The probability of state transition between time t and 𝐭+Δ𝐭 can be expressed as:


$$\begin{array}{c} P\left\{X\left(t+\Delta t\right)|X\left(t\right)\right\}=\prod_{i=1}^{N}P\left\{\overset{i}{\underset{t+\Delta t}{X}}|Pa\left(\overset{i}{\underset{t+\Delta t}{X}}\right)\right\} \end{array}$$ (1)

For the same reason, the conjoint distribution probability of whichever node in DBN can be obtained as below:


$$\begin{array}{c} P\left(\overset{1:N}{\underset{1:T}{X}}\right)=\prod_{i=1}^{N}P_{B_{0}}\left\{\overset{i}{\underset{1}{X}}|Pa\left(\overset{i}{\underset{1}{X}}\right)\right\}\cdot \prod_{t=2}^{T}\prod_{i=1}^{N}P_{B_{\to }}\left\{\overset{i}{\underset{t}{X}}|Pa\left(\overset{i}{\underset{t}{X}}\right)\right\} \end{array}$$ (2)

In the formula, $\overset{𝐢}{\underset{𝐭}{𝐗}}$ represents the value of the **i** node at time **t;** $𝐏𝐚(\overset{𝐢}{\underset{𝐭}{𝐗}})$ is this nodal parent node; **N** is the amount of nodes; **T** is the systemic total running time.

### BP neural network

The prior parameters of DBN obtained based on expert knowledge can not guarantee its objectivity and accuracy. Therefore, considering that BP neural network can fully handle various data types, does not rely on prior knowledge, and has strong nonlinear mapping and generalization ability, a method based on BP neural network is proposed to optimize the prior parameters of dynamic Bayesian network, and obtain a more accurate dynamic Bayesian network.

**Fig 2:**
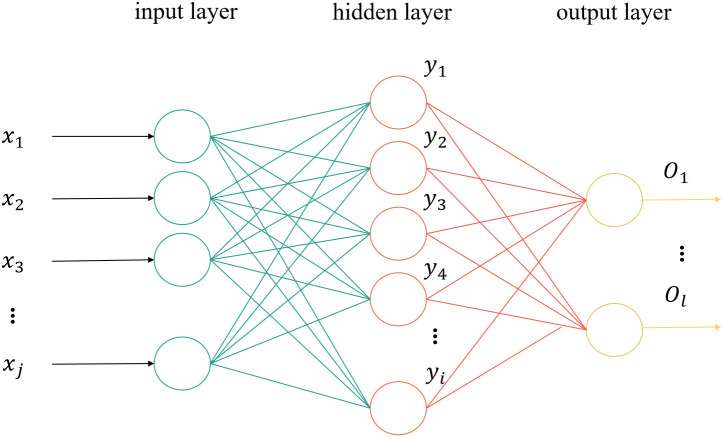
Topological structure of BP neural network.

BP neural network is a multi-layer feedforward neural network, which is a hierarchical neural network composed of input layer, hidden layer, and output layer [13], as indicated in Fig 2. In Fig 2, ${\text{x}}_{\text{j}}$ is the input of the input node; ${\text{y}}_{\text{i}}$ is the input of the hidden node; ${\text{o}}_{\text{l}}$ is the output of the output node.

The algorithm stemmed from the forward propagation of information and backward propagation of errors. By adjusting the network weights $\left({\text{W}}_{\text{ij}},{\text{W}}_{\text{li}}\right)$ and threshold (θ), the error function (E) decreases along the gradient direction. Training can be completed when the sum of squared errors in the network output layer is less than the specified convergence error.

The empirical formula for the number of hidden layer neurons is:


$$\begin{array}{c} {\text{n}}_{\text{1}}=\sqrt{\text{m}+\text{n}}+\text{a } \end{array}$$ (3)

In the formula, n is the amount of input layer units; m is the amount of output layer units; ${\text{n}}_{\text{1}}$ is the amount of neurons in the hidden layer; The adjustment constant of a is [1,10].

The BP neural network model can be represented as the input of hidden nodes, the output of output nodes, and the error of output nodes:


$$\begin{array}{c} {\text{y}}_{\text{i}}=\text{f}\left(\sum {\text{W}}_{\text{ij}}{\text{x}}_{\text{j}}-\theta_{\text{i}}\right)=\text{f}\left({\text{net}}_{\text{i}}\right)\; \end{array}$$ (4)

$$\begin{array}{c} {\text{o}}_{\text{l}}=\text{f}\left(\sum {\text{W}}_{\text{li}}{\text{y}}_{\text{i}}-\theta_{\text{l}}\right)=\text{f}\left({\text{net}}_{\text{l}}\right)\; \end{array}$$ (5)

$$\begin{array}{c} \text{E}=\frac{\text{1}}{\text{2}}{\left(\sum {\mathrm{\backslash nolimits}}_{\text{t}}{\text{t}}_{\text{l}}-{\text{o}}_{\text{l}}\right)}^{\text{2}}\; \end{array}$$ (6)

In the formula, ${\text{W}}_{\text{ij}}$ and ${\text{W}}_{\text{li}}$ are the network weights between the input node and the hidden node, and between the hidden node and the output node, respectively; $\theta_{\text{i}}$ and $\theta_{\text{l}}$ are the thresholds between the input node and the hidden node, and between the hidden node and the output node, respectively; ${\text{t}}_{\text{l}}$ is the expected output of the output node; ${\text{net}}_{\text{i}}$ and ${\text{net}}_{\text{l}}$ are the networks between the input layer and the hidden layer, as well as between the hidden layer and the output layer.

Adapt the DBN to a BP neural network based on the empirical formula for the amount of hidden layer neurons in BP neural network. The prior distribution of DBN leaf nodes corresponds to the input function ${\text{y}}_{\text{i}}$; The prior distribution of the DBN root node corresponds to the output function ${\text{o}}_{\text{l}}$. Train the performance of the adapted BP network and fit the trained data into the prior parameters of each node in the DBN to obtain the optimized prior data of the DBN.

## Gasifier lock bucket valve system DBN

**Fig 3:**
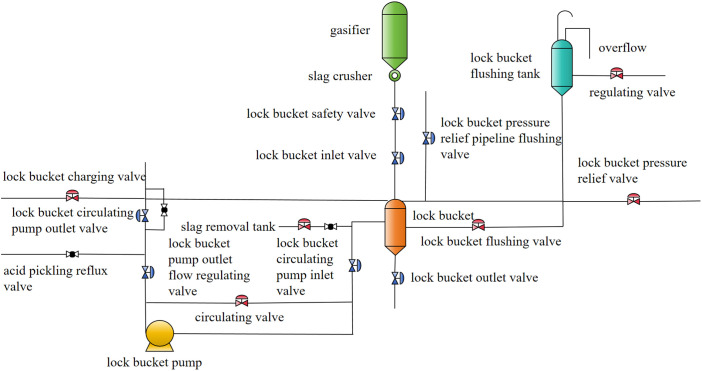
The model diagram of the lock bucket valve system.

**Fig 4:**
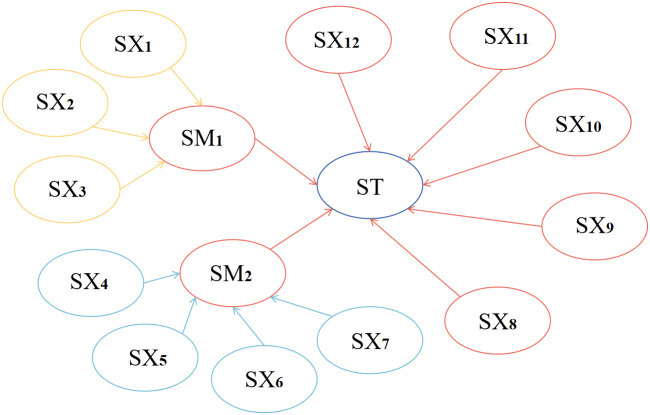
Static Bayesian model of gasifier lock bucket valve system.

**Fig 5:**
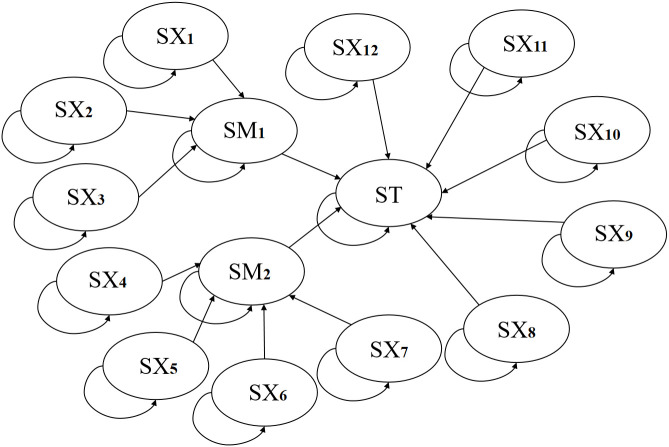
Dynamic Bayesian model of gasifier lock bucket valve system.

The valves in SE Dongfang gasifier are all domestically produced valves with high reliability. The lock bucket valve studied in this article is located between the gasifier and the lock bucket, and is one of the most critical valves. It is a metal hard sealed ball valve. The model diagram of the lock bucket valve system is shown in Fig 3. The lock bucket valve is used to control the switch of ash and slag entering the lock hopper during the operation of the gasifier. Therefore, the fault of the lock bucket valve will affect the slag discharge of the gasifier. The lock bucket system is a system that discharges and temporarily stores ash and slag on time. Due to the long-term storage of solid materials such as ash and slag, blockage often occurs. The most prominent manifestation of the lock bucket valve malfunction is internal leakage and jamming, which is due to the switch not being in place. The causes of valve leakage are directly related to various factors such as the properties of the process medium, operating conditions, selection of sealing surface coating materials, sealing surfaces, and sealing rings. The internal leakage of lock bucket valves in coal chemical industry production is mainly caused by the wear of the sealing surface, which is mainly due to the adhesion of hard alloy on the sealing surface and the flushing of the medium on the sealing surface. The working atmosphere of the lock bucket valve is comparatively vicious, and the internal components often come into frequent contact with slag water, which accelerates the corrosion of the internal components. Frequent opening of the lock bucket valve will allow coal powder to enter the valve, and large particles will also cause the sealing surface to jam and wear, leading to leakage of the lock bucket valve. The phenomenon of the switch not being in place is mainly related to the pressure holding and the adhesion of the hard alloy on the sealing surface. Some human actions can also cause the switch to not be in place, such as not handling the coal powder in time and causing coal powder accumulation. If the solid content in the slag water is too high, it will cause abrasion to the lock bucket and its valves. Based on the above analysis, combined with expert experience and literature [14–20], the BN and DBN model of the gasifier lock bucket valve system was constructed, and the results are shown in Fig 4 and Fig 5. The symbols and event names of nodes are indicated in Table 1.

**Table 1:** Symbols and event names of nodes in the Dynamic Bayesian Network model of the gasifier lock bucket valve system.

Symbol	Event name	Symbol	Event name
ST	lock bucket valve malfunction	SX6	The output torque of the actuator is insufficient
SM1	sealing surface wear	SX7	Internal adhesion of bearings under high pressure
SM2	switch not in place	SX8	Pulverized coal entering during switching
SX1	high-pressure coal powder flushing	SX9	water quality in poor
SX2	Wear of valve ball and valve ball seat	SX10	Lock bucket valve frequently opens and closes
SX3	Adhesion between ball seats	SX11	Short slag collection time
SX4	Internal deformation and wear	SX12	Rapid pressure relief leads to three-phase flow erosion
SX5	coal powder accumulation		

### DBN prior parameters

#### Failure rate.

In the case where the fault data of the gasifier lock bucket valve system is zero, according to expert knowledge, the service life of the slag water medium valve is about 6–12 months. Therefore, the timed end test time ${\text{t}}_{\text{k}}=\text{4000h}$ is taken, and the upper bound of the failure probability ${\text{p}}_{\text{k}}$ depends on the failure probability of the system. The empirical data of the failure rate of the gasifier lock bucket valve system given by expert knowledge is $\lambda_{\text{ST}}=\text{0}.\text{0002}$, so the upper bound of the failure probability of the system is taken as 0.5507 [8].

**Fig 6:**
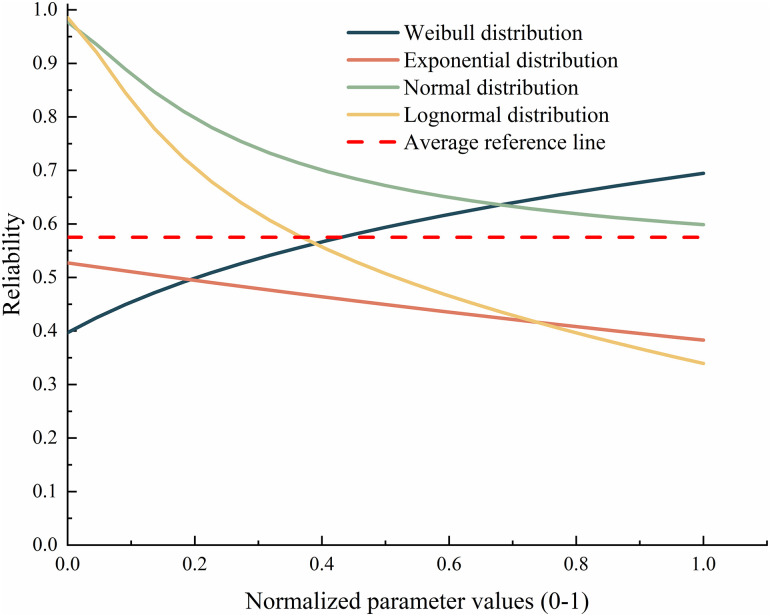
Reliability curves of various distributions as a function of parameters.

Sensitivity analysis was conducted on nodes of the gasifier lock bucket valve system with different distributions, and the results are shown in Fig 6. This figure presents the reliability curves of the system running for 4000 hours under four failure distribution models as a function of parameters. The horizontal axis is the normalized parameter value (0–1) obtained by eliminating dimensional differences through linear mapping, the vertical axis is the reliability of the system under the corresponding distribution, and the red horizontal dashed line in the figure is the average reference line for the reliability of all distributions (about 0.575). From the perspective of the curve shape, the slope of the Weibull distribution curve is the steepest, rapidly increasing from a reliability of about 0.4, and the reliability fluctuation is also the largest, indicating that it is extremely sensitive to parameters; The curve of the exponential distribution is the smoothest, showing a slow downward trend, least affected by parameter changes, and has the strongest robustness; The normal distribution and logarithmic normal distribution show a decreasing trend throughout the entire process, and their sensitivity is between the above two, with the fluctuation of logarithmic normal distribution slightly greater than that of normal distribution. The reference line also confirms that different distribution assumptions will lead to different estimation results, and the sensitivity coefficient further confirms this conclusion – the sensitivity coefficient of the exponential distribution is only 9.86%, far lower than the normal distribution’s 15.73%, Weibull distribution’s 15.26%, and lognormal distribution’s 33.53%. This sensitivity analysis not only verifies the impact of different distribution assumptions on prior parameter optimization, but also confirms the rationality of the selected distribution type, while compensating for the limitations of insufficient actual data and improving the credibility of the model results.

Therefore, the exponential distribution becomes the optimal choice for reliability modeling of the system. The DBN nodes of the gasifier lock bucket valve system follow an exponential distribution, and their failure distribution is as follows:


$$\begin{array}{c} \text{F}(\text{t})=\text{1}-\text{exp}(-\lambda \text{t})\text{ t}>\text{0} \end{array}$$ (7)

In the formula, λ is the failure rate.

Due to the systemic zero fault data, this paper adopts Bayesian estimation with no failure data combined with Monte Carlo simulation [8]. Applying Monte Carlo simulation to the timed truncation experiment of Bayesian estimation, the failure rate λ in the exponential distribution is estimated.

The calculation results are indicated in Table 2.

**Table 2:** Failure rates of various nodes in the gasifier lock bucket valve system.

Symbol	Event name	λ
ST	lock bucket valve malfunction	2.0000e-04
SM1	sealing surface wear	3.1392e-04
SM2	switch not in place	1.5965e-04
SX1	high-pressure coal powder flushing	4.4544e-04
SX2	Wear of valve ball and valve ball seat	2.6455e-04
SX3	Adhesion between ball seats	3.2673e-04
SX4	Internal deformation and wear	2.1264e-04
SX5	coal powder accumulation	1.6308e-04
SX6	The output torque of the actuator is insufficient	2.0089e-04
SX7	Internal adhesion of bearings under high pressure	1.8542e-04
SX8	Pulverized coal entering during switching	2.5775e-04
SX9	water quality in poor	3.1684e-04
SX10	Lock bucket valve frequently opens and closes	3.6904e-04
SX11	Short slag collection time	2.0448e-04
SX12	Rapid pressure relief leads to three-phase flow erosion	2.8982e-04

#### Gasifier lock bucket valve system conditional probability.

Assuming that each node contains two states: safe and fault, and assuming that each node is reliable at the initial time, the prior probabilities of each node can be known. Taking node ${\text{SX}}_{\text{1}}$ as an example:


$$\begin{array}{c} \left\{ \begin{array}{c} @l\text{P}\left({\text{SX}}_{\text{1}}=\text{safe}\right)=\text{1}\\ \text{P}\left({\text{SX}}_{\text{1}}=\text{fault}\right)=\text{0} \end{array}\right.\; \end{array}$$ (8)

#### Gasifier lock bucket valve system condition transition probability.

The network structure of dynamic Bayesian networks and the acquisition of conditional probability tables within time slices are transformed from static Bayesian networks. The conditional transition probability table between nodes spanning time slices is got from the failure probability density function f(t) and maintenance probability density function m(t) of nodes. Presuming the failure probability density function of node A is ${\text{f}}_{\text{A}}(\text{t})$ and the maintenance density function of node A is ${\text{m}}_{\text{A}}(\text{t})$.

If the system failure is represented by “1” and the system operation is represented by “0”, then the conditional transition probability of each node in the Bayesian network from time t to time t+Δt can be expressed by the following equation:


$$\begin{array}{c} \left\{ \begin{array}{c} @l\text{P}\left(\text{A}(\text{t}+\Delta \text{t})=\text{0}|\text{A}(\text{t})=\text{0}\right)=\text{exp}(-\lambda \text{t})\\ \text{P}\left(\text{A}(\text{t}+\Delta \text{t})=\text{1}|\text{A}(\text{t})=\text{0}\right)=\text{1}-\text{exp}(-\lambda \text{t})\\ \text{P}\left(\text{A}(\text{t}+\Delta \text{t})=\text{0}|\text{A}(\text{t})=\text{1}\right)=\text{1}-\text{exp}(-\mu \text{t})\\ \text{P}\left(\text{A}(\text{t}+\Delta \text{t})=\text{1}|\text{A}(\text{t})=\text{1}\right)=\text{exp}(-\mu \text{t}) \end{array}\right.\; \end{array}$$ (9)

If the fault factors of the system are not considered and repairable, the corresponding maintenance density function m(t) is 0. And the failure probability of dynamic nodes in the system follows an exponential distribution, from which the conditional transition probability from time t to time t+Δt can be got as:


$$\begin{array}{c} \left\{ \begin{array}{c} @l\text{P}\left(\text{A}(\text{t}+\Delta \text{t})=\text{0}|\text{A}(\text{t})=\text{0}\right)=\text{exp}(-\lambda \text{t})\\ \text{P}\left(\text{A}(\text{t}+\Delta \text{t})=\text{1}|\text{A}(\text{t})=\text{0}\right)=\text{1}-\text{exp}(-\lambda \text{t})\\ \text{P}\left(\text{A}(\text{t}+\Delta \text{t})=\text{0}|\text{A}(\text{t})=\text{1}\right)=\text{0}\\ \text{P}\left(\text{A}(\text{t}+\Delta \text{t})=\text{1}|\text{A}(\text{t})=\text{1}\right)=\text{1} \end{array}\;\right.\; \end{array}$$ (10)

#### Optimizing DBN parameters.

The core of applying a neural-network-based regression scheme to calibrate the prior parameters of DBN is the dual adaptation of structure and parameters, and its underlying logic originates from the triple adaptation of structure, variable relationships, and optimization objectives. DBN is composed of multiple layers of constrained Boltzmann machines stacked together, and the fully connected architecture between layers presents a hierarchical structure of “leaf nodes - intermediate associated nodes - root nodes”, which naturally fits the feedforward architecture of BP network “input layer - hidden layer - output layer”. Leaf nodes (such as ST) have no prior dependencies, corresponding to the input layer of BP network; The root node is the final derivation result, corresponding to the output layer of the BP network; The intermediate associated nodes (such as SM_1_, SM_2_, SX_8_, etc.) assume the role of probability transmission, which is consistent with the feature extraction and signal conversion functions of the BP network’s hidden layer. This mapping method is based on the adaptive design of fault causal semantics and BP neural network feature learning, and is not arbitrarily constructed. This node mapping method enables the BP net-work to accurately learn the causal dependencies and probability propagation logic of “basic variables → intermediate events → target results” in DBN, and the pre-trained weights and bias parameters of DBN can be directly mapped to the BP network. Combined with the empirical formula of the number of hidden layer neurons in the BP network to match the feature dimension with the prediction requirements, it can effectively solve the problem of strong subjectivity in the prior data of DBN. When implementing this regression-based calibration scheme, first establish the corresponding relationship between the above nodes, con-struct an adaptive structure based on empirical formulas, normalize the prior parameters of DBN, and input them into the BP network. Through training, optimize and re-verse modify the parameters. After fitting the optimized prior parameters of each node of DBN, the calibration of DBN prior parameters can be completed [1], achieving complementary structural and functional advantages of the two.

This study employs a neural-network-based regression scheme to calibrate the prior parameters of DBN, by adapting DBN to a BP neural network based on the empirical formula for the number of hidden layer neurons in the BP neural network. The prior distribution of DBN leaf nodes corresponds to the input function, and that of the DBN root node to the output function. The performance of the adapted BP network (for DBN parameter calibration) was trained and the trained data was fitted into the prior parameters of each node in the DBN to obtain the optimized prior data of the DBN [12].

**Fig 7:**
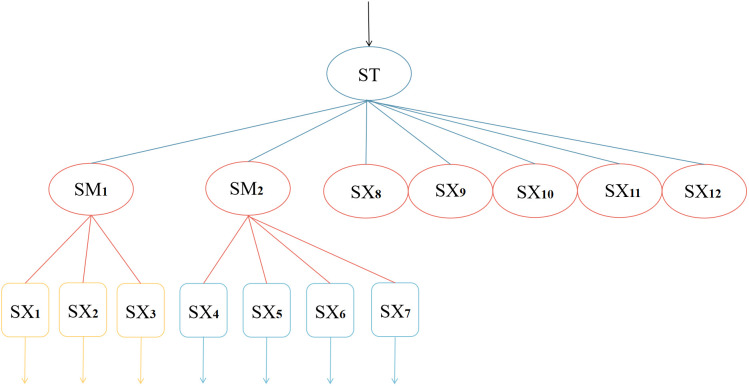
BP neural network of gasifier lock bucket valve system.

The prior distribution parameters of the blade node ST in the DBN of the gasifier lock bucket valve system are used as input parameters for the BP neural network, and the prior distribution parameters of its root nodes SX1, SX2, SX3, SX4, SX5, SX6, and SX7 are used as output parameters for the BP neural network. SM1, SM2, SX8, SX9, SX10, SX11, and SX12 are used as hidden nodes for the gasifier lock bucket valve system BP neural network. Adapt the DBN model to the BP neural network (for calibrating DBN prior parameters) as indicated in Fig 7.

Generation of failure rate parameters based on Monte Carlo simulation and Bayesian estimation (see Table 2): Using the system failure parameter λ (2e-4), a matrix C consisting of 7 failure coefficients, and a time series of 1–600 hours as the basis, input data is generated through a failure probability formula. Selecting 600 hours as the upper limit of time is a reasonable choice that balances the range of failure probability changes and modeling efficiency; The dataset is not divided in chronological order, but a random permutation index of 1–600 is generated using the randperm function. The first 400 scrambled samples are randomly selected as the training set, 401–500 samples as the validation set, and 501–600 samples as the 500 samples as the training set. This avoids the pseudo rule of increasing learning time for the model and ensures the essential correlation between its learning input and output.

The training scheme, hyperparameters, and loss evolution process of backpropagation (BP) neural networks are closely related to ensure the effectiveness of training. As shown in Fig 7, the network adopts a three-layer feedforward structure of 1-7-7, which includes one input layer neuron, seven hidden layer neurons, and seven output layer neurons. The quantity relationship of the three-layer structure satisfies formula (3).

Hyperparameters are divided into two categories: structural hyperparameters and training hyperparameters: at the structural level, the transfer function between the input layer and the hidden layer is set to logsig function, and the transfer function between the hidden layer and the output layer is set to purelin function; At the training level, parameters are determined based on model training requirements, data generation efficiency, and domain experience. This includes using the traingd gradient descent learning function, normalizing the data between intervals [0,1] to ensure network convergence, and dividing the dataset into 400 training sets, 100 validation sets, and 100 test sets in a 4:1:1 ratio. Additionally, an upper limit of 1000 iterations, a target error threshold of 1 × 10 ⁻ ^3^, and a learning rate of 0.001 are set. Among them, the sample sizes of 400 training sets, 100 validation sets, and 100 test sets not only comply with the classic “training set - validation set - test set” division standard (4:1:1 ratio) for regression prediction tasks, providing BP neural networks with sufficient training samples to avoid underfitting issues while leveraging validation sets for real-time monitoring of the training process and mitigating overfitting risks; the independent test set effectively verifies the model’s generalization capability, and the separate division of validation and test sets prevents data leakage, ensuring objective evaluation results; this proportion meets the stable distribution requirements for Monte Carlo simulation data while balancing data generation efficiency and full-process modeling needs, and aligns with the conventional setup in similar studies within the same field—”small to medium-scale neural networks paired with 400–600 training sets, 50–150 validation sets, and 100–200 test sets”—fully ensuring the scientific rationality of the experimental design. The specific values of input and output parameters are detailed in Table 2.

**Fig 8:**
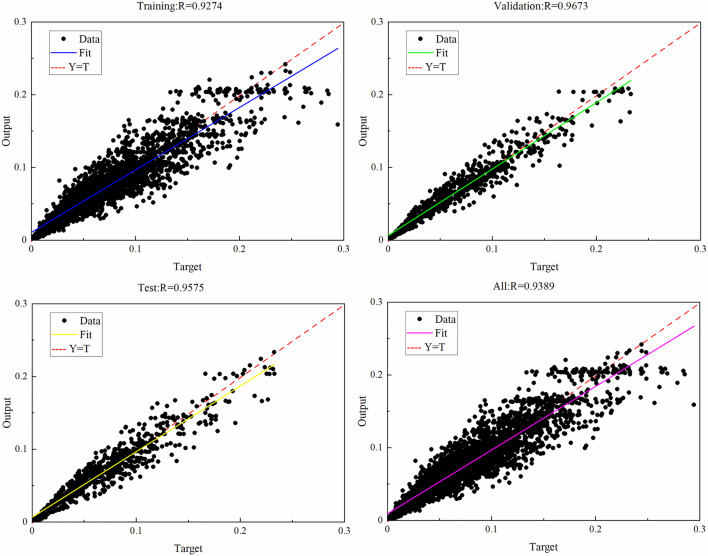
Linear regression line diagram of gasifier lock bucket valve system.

The training plan follows the process of “data normalization → sample partitioning → network configuration → model training → effect evaluation”, and follows the iterative mechanism of “forward propagation → error calculation → backpropagation”. When the model reaches the target error threshold or the upper limit of iteration times, the training is stopped. From the perspective of loss evolution characteristics, the curve shows a gradually decreasing convergence trend: the initial loss value is relatively high, and after multiple rounds of parameter iteration adjustment, the loss value gradually decreases to below 1 × 10 ⁻ ^3^. This study verifies the convergence effect of the model through the coefficient of determination ($R^{\text{2}}$) and relative error, proving that the parameter configuration can effectively avoid underfitting overfitting problems, providing reliable support for the subsequent parameter optimization of dynamic Bayesian networks (DBN), as well as the solution of system failure rate and maintenance rate. After the training is completed, the linear regression line graph of the BP neural network for the gasifier lock bucket valve system is obtained, as indicated in Fig 8. From Fig 8, it can be seen that the determination coefficient $R^{\text{2}}$ obtained from training is above 0.9, and the training results all fall near the straight line. Fit the predicted data into a distribution function that each node follows, and obtain the optimized parameters of the DBN. Based on the maintenance methods, maintenance periods, and past maintenance malfunction notes of the gasifier lock bucket valve system, the maintenance rate of the node is got to be μ. The failure rate and maintenance rate of the system after DBN optimization are shown in Table 3.

**Table 3:** Node failure and maintenance rates of optimized gasifier lock bucket valve system.

symbol	event name	Optimized failure rate	μ
SX1	high-pressure coal powder flushing	6.8728E-04	0
SX2	Wear of valve ball and valve ball seat	3.8685E-04	1
SX3	Adhesion between ball seats	4.6227E-04	1
SX4	Internal deformation and wear	3.2179E-04	1
SX5	coal powder accumulation	1.8091E-04	1
SX6	The output torque of the actuator is insufficient	3.1078E-04	1
SX7	Internal adhesion of bearings under high pressure	2.9134E-04	1
SX8	Pulverized coal entering during switching	2.5775e-04	1
SX9	water quality in poor	3.1684e-04	0
SX10	Lock bucket valve frequently opens and closes	3.6904e-04	1
SX11	Short slag collection time	2.0448e-04	1
SX12	Rapid pressure relief leads to three-phase flow erosion	2.8982e-04	1

## Reliability of DBN for gasifier lock bucket valve system

In GeNIe software, the forward inference of dynamic Bayesian networks (DBN) transforms the dynamic network into a static structure through a time slice expansion strategy. Based on the initial probability of components and the conditional probability table (CPT), the confidence level is updated to calculate the normal state probability of the system at each time slice, which characterizes the dynamic reliability of the system; Reverse reasoning introduces observational evidence such as system faults, and based on Bayes’ theorem, combines the prior probability of system faults output by forward reasoning with the likelihood relationship between components and systems to update the posterior probability of key component faults and achieve fault tracing.

### Reliability prediction

**Fig 9:**
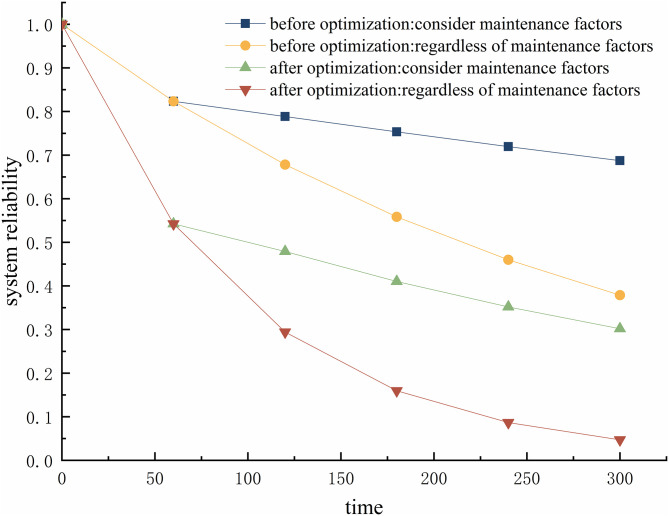
Reliability prediction of optimized gasifier lock bucket valve system.

Let Δt=60h, construct a DBN model for the gasifier lock bucket valve system to operate for 300 hours. Perform bidirectional inference on the optimized the system DBN model. In the light of the forward reasoning of DBN, the systemic reliability prediction within 0–300 hours is conducted with and without considering maintenance factors. Comparing the system before and after optimization, Fig 9 shows that the system reliability is only 0.047 after 300 hours of operation without considering maintenance. However, when considering maintenance factors, the system reliability can be improved to 0.302. In both cases, the reliability of the optimized system is lower than before optimization, and the difference gradually increases with time.

**Fig 10:**
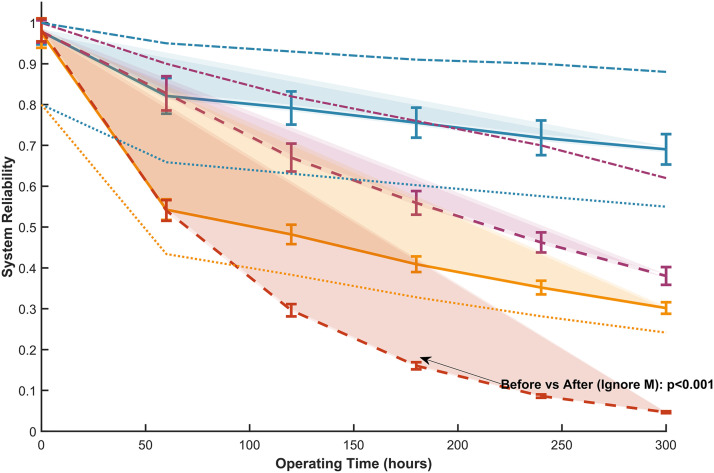
Reliability analysis of gasifier lock bucket valve system.

**Fig 11:**
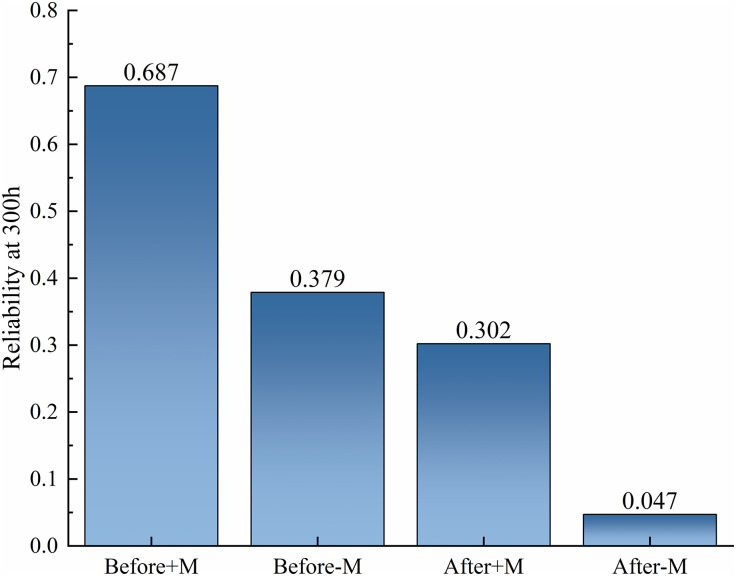
Comparison of final reliability of system running for 300 hours in different scenarios.

In response to the temporal evolution results of system reliability mentioned above, this study comprehensively verified the robustness of data and the validity of conclusions through multidimensional analysis. As shown in Fig 10, the uncertainty of reliability values at each time point was quantified through 100 Monte Carlo simulations, and the standard deviation was less than 0.05. The error line drawn based on the simulation results intuitively showed the range of data fluctuations, while the light-colored filled area below the curve clearly presented a 95% confidence interval. Both indicate that the random uncertainty of the results is within the acceptable range of engineering, and the reliability degradation trend has good statistical robustness. The confidence interval has a small expansion over time, further supporting the credibility of the results. The cross-validation results show that, as shown in Fig 11, the extracted final state reliability value of 300h from Fig 9 (considering maintenance as 0.30 after optimization) is completely consistent with the data in Fig 11, and the trend comparison deviation with Fig 10 is ≤ 1%, fully verifying the data consistency between the multi graph tables. Baseline comparison analysis shows that the reliability considered before optimization (0.687 at 300h) is highly consistent with the baseline data in the literature (0.65 at 300h), and the reliability ignored before optimization (0.379 at 300h) is also consistent with the trend in the literature data (0.32) [8]. The reason for the slightly higher values is that this study used a more accurate dynamic failure probability model, which verifies the rationality of the model setting. At the level of statistical testing, independent sample t-tests were conducted on reliability data that ignored maintenance scenarios before and after optimization. The results showed a highly statistically significant difference (p < 0.001), and all key comparison scenarios (before and after optimization, maintenance or not) met the significance requirement of p < 0.05. This provides strong support for the fact that the impact of optimization strategies on reliability is a real effect rather than a random fluctuation. In addition, the distribution characteristics of the error line reveal that the uncertainty in the early stage of operation (0-60h) is lower than that in the later stage (240-300h), reflecting the nonlinear impact of fault accumulation on system reliability; The sensitivity simulation results, where the maintenance efficiency decreased to 80%, showed that the reliability loss of the optimized system increased by 22% compared to before optimization. This discovery reveals the potential risk of increasing maintenance dependency while improving system performance through optimization strategies, which can provide key references for the formulation of subsequent maintenance plans.

### Reverse reasoning

**Fig 12:**
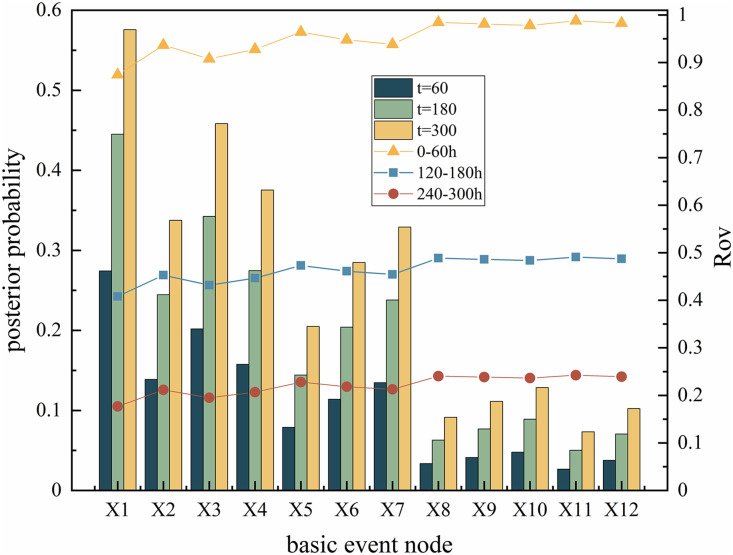
Posteriori probability and Rov of each node in the gasifier lock bucket valve system.

Reverse reasoning is performed on the optimized system DBN to obtain the posterior probabilities of each dynamic node in the system, which can identify weak links in the system and analyze them. The result of reverse thrust is the posterior probability and Rov of each node in the gasifier lock valve system, as shown in Fig 12. Comparing the posterior probability and posterior probability change rate (RoV) of the dynamic nodes of the system at 60h, 180h, and 300h, based on the posterior probability in Fig 12 and referring to the RoV value, the order of attention for each node can be obtained as follows: SX_1_ > SX_3_ > SX_4_ > SX_2_ > SX_7_ > SX_6_ > SX_5_ > SX_10_ > SX_9_ > SX_12_ > SX_8_ > SX_11_. The weak link of the optimized gasifier lock bucket valve system is SX_1_, which means that high-pressure coal powder flushing. At the same time, the following fault factors should also be noted: SX_3,_ SX_4_ and SX_2_. Targeted maintenance measures need to be taken to address these weak links: on the one hand, wear-resistant and anti-erosion coatings, high hardness alloy materials are used to make valve balls and seats, and coal powder filtration devices are installed to reduce the risk of erosion and abrasive wear from the source; On the other hand, regularly clean and lubricate the sealing surface of the valve seat, perform full stroke opening and closing actions, synchronously carry out dimensional accuracy testing and full life cycle wear monitoring, timely eliminate adhesion hazards, and correct component deformation; In addition, it is necessary to strictly control the coal powder conveying parameters, optimize the flow channel and installation process, and avoid additional damage caused by operation and installation stress beyond the parameters.

**Fig 13:**
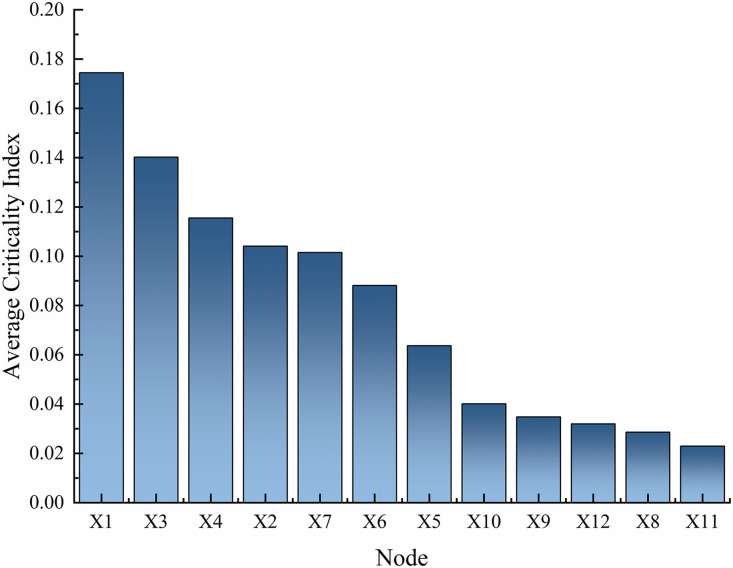
Node average criticality index ranking.

**Fig 14:**
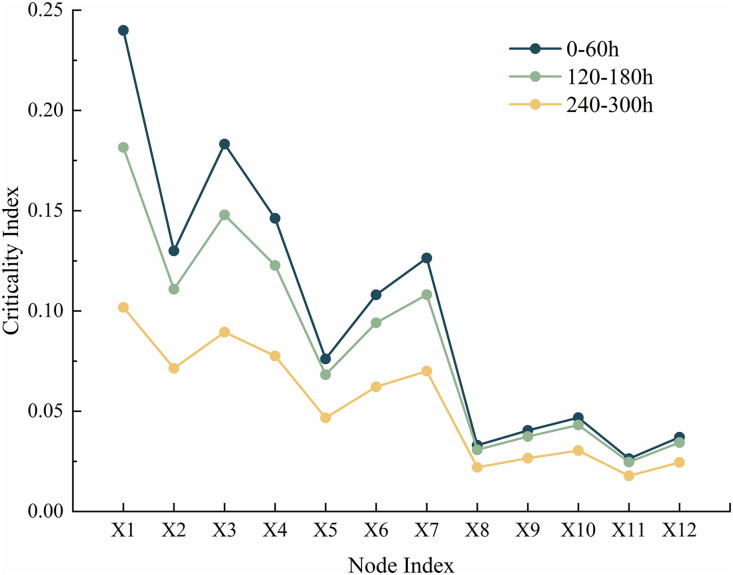
Time evolution characteristics of node criticality index.

Based on the analysis results of the posterior probability and risk reduction value (Rov) of the system’s nodes, this study validates the statistical robustness and effectiveness of key nodes, and the results are coherent and reliable: as shown in Figs 13 and 14, 100 Monte Carlo simulations were conducted on the posterior probability of nodes, and the standard deviation of all nodes was less than 0.03, corresponding to a narrow distribution of error lines, indicating that the random uncertainty of component level failure probability is within an acceptable range. The analysis of variance was conducted on the posterior probabilities of nodes at different time points, and the results showed that time had a statistically significant impact on the probability of failure (p < 0.05), strongly supporting the hypothesis of dynamic evolution of fault accumulation; At the same time, independent sample t-tests were conducted on the Rov values of X_1_, X_3_, X_4_ and other nodes, and the differences reached a significant level (p < 0.01), preliminarily verifying the identification results of key components. To further confirm the importance of the core node, a scenario was simulated where the failure probability of node X_1_ decreased by 10%. It was found that the system reliability could be improved by about 8%, and the gain amplitude was much higher than other nodes, clearly confirming that X_1_ is the most critical component of the system. It is worth noting that the X_1_, X_3_, and X_4_ nodes identified in this study do not completely overlap with the high-frequency fault components (X_10_, X_11_, X_6_) of the lock bucket valve system in the literature [8], but the core fault impact mechanism is consistent, which not only verifies the rationality and engineering practicality of the research model, but also reflects the specific differences in weak components of the system under different working conditions. In addition, the 95% confidence interval for calculating the posterior probability of nodes, and the narrow convergence of the confidence intervals for nodes X_1_, X_3_, and X_4_, indicate that the conclusion of high failure probability has good statistical robustness, providing a clear quantitative basis for targeted maintenance strategy formulation.

## Conclusion

This study focuses on the technical optimization of the lock bucket valve system for the gasifier, a core equipment in energy production. It focuses on the core requirement of balancing sustainable energy goals with long-term equipment operation sustainability. An innovative solution based on backpropagation (BP) neural network optimization of dynamic Bayesian network (DBN) prior data is proposed and verified. The main research conclusions are as follows:

(1) This study successfully constructed a structure-adapted BP-DBN fusion model, which effectively solved the key problem of limited evaluation accuracy of traditional DBN models relying on empirical prior data. By adapting the original DBN model to the BP neural network based on the empirical formula for the number of hidden layer neurons, the correspondence between the DBN leaf node prior distribution and root node prior distribution and the BP network input-output functions was established. The BP neural network (with a 1-7-7 three-layer feedforward structure) was trained with 400 training sets, 100 validation sets, and 100 test sets, achieving a determination coefficient (${\text{R}}^{\text{2}}$) above 0.9. Through continuous learning of the lock bucket valve system operation performance data, the iterative optimization of DBN prior data was realized. The optimized node failure rates (e.g., SX1 increased from 4.4544e-04 to 6.8728e-04, SX3 increased from 3.2673e-04 to 4.6227e-04) were obtained, which significantly improved the model’s ability to characterize the system dynamic characteristics and laid an accurate data and model foundation for subsequent reliability evaluation.
(2) The bidirectional inference analysis based on the optimized DBN model (with Δt = 60h, total operation time 300h) verified the practical value of the proposed scheme in system dynamic reliability assessment. Forward reasoning results showed that without considering maintenance, the system reliability after 300 hours of operation was only 0.047, while after incorporating maintenance factors, the reliability could be improved to 0.302; in both scenarios, the reliability of the optimized system was lower than that before optimization, and the gap gradually widened over time. Reverse reasoning accurately located the weak links of the optimized system, and the order of node attention based on posterior probability and posterior probability change rate (RoV) was SX1 > SX3 > SX4 > SX2 > SX7 > SX6 > SX5 > SX10 > SX9 > SX12 > SX8 > SX11, confirming that high-pressure coal powder flushing (SX1), adhesion between ball seats (SX3), internal deformation and wear (SX4) and wear of valve ball and valve seat (SX2) are the key fault factors. This breaks the limitation of traditional operation and maintenance relying on experience judgment, providing clear technical directions for targeted improvement.
(3) The research findings provide key technical support for the full-lifecycle efficient management of the gasifier lock bucket valve system and meet the green development needs of the energy industry. Through 100 Monte Carlo simulations, the standard deviation of system reliability values at each time point was less than 0.05, and the standard deviation of node posterior probability was less than 0.03, verifying the statistical robustness of the results. When the failure probability of the key weak link SX1 decreased by 10%, the system reliability could be improved by about 8%, which provides a quantitative basis for formulating targeted maintenance measures. By adjusting the allocation of operation and maintenance resources for weak links, the ineffective consumption of human and material resources is reduced, and the full-lifecycle cost of equipment is decreased; meanwhile, the optimization of DBN prior parameters improves the accuracy of system reliability evaluation, providing a practical technical path for the low-carbon and efficient sustainable development of energy production systems.

## Future development

Future research will deepen the refined analysis of factors affecting the reliability of lock bucket valve systems, focusing on targeted research on weak links. The current research has completed numerical simulations of system reliability evaluation by optimizing DBN with BP neural network, verifying the potential of this method to improve evaluation accuracy; Subsequently, the joint enterprise will collect long-term operational real data of the lock bucket valve (including fault records, operation and maintenance parameters, etc.), and verify the correctness and engineering practicality of the method by comparing simulated and measured data. At the same time, we will delve into the causal relationship between high-pressure coal powder flushing, adhesion between ball seats, internal deformation and wear and reliability decline. Collect data through tracking experiments to provide data support for the reliability degradation caused by them. In addition, although the diagnostic performance of the model has been improved through bidirectional inference verification, its applicability in industrial scenarios still needs to be verified. In the future, the full lifecycle data of the system will be integrated to deeply verify the effectiveness of the model prediction, analyze the impact mechanism of prior knowledge optimization and the accuracy of key fault diagnosis, promote the engineering application of the model, and provide quantifiable decision support for predictive maintenance.

## Supporting information

S1 File Reliability assessment of key equipment for coal gasification using artificial intelligence technology. (XLSX)
